# TDP-43 and HERV-K Envelope-Specific Immunogenic Epitopes Are Recognized in ALS Patients

**DOI:** 10.3390/v13112301

**Published:** 2021-11-18

**Authors:** Elena Rita Simula, Giannina Arru, Ignazio Roberto Zarbo, Paolo Solla, Leonardo A. Sechi

**Affiliations:** 1 Department of Biomedical Sciences, University of Sassari, Viale San Pietro 43b, 07100 Sassari, Italy; simulaelena@gmail.com; 2 Department of Clinical, Surgical and Experimental Sciences, University of Sassari, Viale San Pietro 8, 07100 Sassari, Italy; giarru@uniss.it (G.A.); irzarbo@uniss.it (I.R.Z.); psolla@uniss.it (P.S.); 3 Struttura Complessa Microbiologia e Virologia, Azienda Ospedaliera Universitaria, 07100 Sassari, Italy

**Keywords:** ALS, amyotrophic lateral sclerosis, TDP-43, HERV-K, HML-2

## Abstract

The human endogenous retrovirus-K (HERV-K) and TAR DNA-binding protein 43 (TDP-43) have been associated with the pathophysiology of amyotrophic lateral sclerosis (ALS). Given these findings, we investigated the humoral response against HERV-K envelope surface (env-su) glycoprotein antigens and TDP-43 in the plasma of ALS patients and healthy controls (HCs). The measured levels of Abs against the different epitopes’ fragments were significantly elevated in ALS patients, both in long-survivor (LS) and newly diagnosed (ND) patients, compared to HCs. We observed a positive correlation between HERV-K and TDP-43 antibodies (Abs) levels, which seemed to strengthen with disease progression, that was not found in HCs. The TDP-43 and HERV-K epitopes identified in this study are highly immunogenic and recognized by the humoral response of ALS patients. Increased circulating levels of Abs directed against specific HERV-K- and TDP-43-derived epitopes could serve as possible biomarkers.

## 1. Introduction

Amyotrophic lateral sclerosis (ALS) is a neurodegenerative disease of unknown etiology described for the first time in 1874 by Jean-Martin Charcot. It is characterized by the progressive degeneration of both the upper and lower motor neurons, which display cytoplasmatic inclusions [1,2]. Population-based studies have highlighted that women are less affected by sporadic ALS (sALS) than men, while the same incidence between men and women in familial ALS (fALS) is observed. The incidence decreases rapidly after 80 years of age, whereas the 58–63 and 47-52 age brackets represent the peak ages of onset for sALS and fALS, respectively [3]. The ALS condition is primarily characterized by the involvement of the pyramidal tract with the progressive reduction in upper motor neuron activity, which originates from the motor cortex, and lower motor neurons, which connect the spinal cord and brainstem to skeletal muscles. Common features of ALS are progressive muscle weakness and atrophy, fasciculations, dysarthria, and dysphagia. Remarkably, a significant proportion of cases presents frontotemporal lobe degeneration (FTLD) with cognitive damage. Respiratory complications are the final stage, after which death occurs within two to five years of diagnosis [4]. The main causes of ALS are still unknown, but significant advances have been made in understanding the environmental and genetic components involved in the pathology [5]. There are several hypotheses behind the development of the disease, involving both genetic predisposition, with more than 24 implicated genes, such as Superoxide dismutase 1 (SOD1) and TAR DNA-binding protein 43 (TDP-43) [6], and environmental risk factors, for instance, smoking [7], physical activity [8], and chemical exposure [9,10].

Emerging evidence has indicated that human endogenous retrovirus K (HERV-K) may play a role in the disease’s etiopathogenesis. Human endogenous retroviruses (HERVs), considered until recently as junk DNA, constitute nearly 8% of the human genome. Proviral genome integrations into the DNA are remnants of infections that occurred over several million years ago. Nevertheless, numerous nonsense mutations have contributed to their defection [11].

HERV-K, in particular the HML-2 subgroup, is present in hundreds of copies in the human genome and is one of the most recently acquired and the most transcriptionally active among HERVs. The involvement of HERV-K in the pathophysiology of ALS has been documented by numerous groups. Douville et al., (2011) observed an altered expression of HERV-K *pol* transcripts in post-mortem brain samples of patients affected by ALS compared to other pathological conditions such as Parkinson’s disease, chronic systemic illnesses, and healthy subjects [12]. Transgenic animals, in whose neurons the *env* gene was expressed, developed progressive motor dysfunction [13].

It is noteworthy that HERV-K expression is regulated by TDP-43, which can bind to the long terminal repeat region (LTR) of the virus. Human neurons transfected with TDP-43 showed an increase in HERV-K expression, while knockdown of endogenous TDP-43 resulted in a decrease in HERV-K expression [13]. Furthermore, it was demonstrated that Abs levels against HERV-K-env-su_(20–38)_ were significantly elevated in ALS patients compared to multiple sclerosis (SM), Alzheimer’s disease (AD), and healthy control subjects, both in serum and cerebrospinal fluid (CSF), suggesting HERV-K-env-su_(20–38)_ as a possible early novel biomarker [14]. Specific post-translational modifications of TDP-43 may have an impact on HERV-K expression patterns. Concerning this, the formation of TDP-43 aggregates alters HERV-K RT, polyprotein levels, and the cellular localization of the viral proteins [15]. Li et al. [13] demonstrated that HERV-K LTR has four binding sites for TDP-43 that have been shown to regulate its activation. In HIV patients an increased nuclear TDP-43 expression accompanied by an enhanced TDP-43 phosphorylation compared to the levels observed in controls has been reported. The co-expression of HERV-K RT and TDP-43 proteins has been observed in the majority of neurons with a significant positive correlation between them [16].

TDP-43 is a multifunctional protein associated with several biological functions [17], including mRNA transcription, splicing and stability [18,19,20], stress granule formation [21], and Protein-Protein Interactions [22]. TDP-43 is indispensable for the development of the central nervous system (CNS) from the earliest stages of embryonic life to adulthood [23,24]. Under physiological conditions, TDP-43 is mainly a nuclear protein, but it also shuttles to the cytoplasm for further functions. Neurons transfected with TDP-43 mutants altering nuclear trafficking exhibit cytoplasmic aggregates with phosphorylated and ubiquitinated TDP-43, which is characteristic of ALS pathology [25]. The TARDBP gene is located on chromosome 1, and the TDP-43 protein consists of 414 amino acids. The C-terminal region, which plays a crucial role in the disease, encompasses a prion-like glutamine/asparagine-rich (Q/N) domain and a glycine-rich region [26]. TDP-43 represents a target for numerous post-translational modifications that may change its structure, functions, localization, and its aggregative predisposition [27,28]. The most documented post-translational modifications in ALS patients are phosphorylation in serine-409 and serine-410 [29,30], but other phosphorylated sites of pathological TDP-43 have also been described, such as serine-379, 403, and 404 [29,31,32]. Kametani et al., showed that all serine and threonine residues in the C-terminal domain can become phosphorylated [33], and this may increase the tendency of TDP-43 to be hydrolyzed into C-terminal fragments or to aggregate [34].

To date, the ALS diagnostic process requires approximately one year, since only the progression of symptoms and the presence of signs of both upper and lower motor neuron involvement can confirm the ALS diagnosis. The diagnosis is based on clinical examination, electrophysiological findings, medical history, and exclusion of confounding disorders [35]. Unfortunately, no effective therapies able to cure the disease are available; Riluzole is the only therapy that can prolong ALS survival (by approximately three months). Different treatments are available to help control ALS symptoms, prevent unnecessary complications, and make it easier for patients to live with the disease. This study aims to more deeply understand the possible relationship between TDP-43 and HERV-K through the investigation of the humoral response to different epitopes of the TDP-43 C-termina region and the HERV-K envelope. The detection of circulating autoantibodies is crucial in the diagnosis and monitoring of several diseases, and it helps to understand the etiopathogenesis. Furthermore, this approach may be useful to hypothesize new biomarkers for ALS which would allow a faster diagnosis.

## 2. Materials and Methods

### 2.1. Samples Collection

We evaluated an ALS group of 45 patients (17 females and 28 males; mean age ± SD: 64.67 ± 9.11 years), and an age- and gender-matched healthy control group (HCs) collected from the Blood Transfusion Centre of Sassari (17 females and 28 males; mean age ± SD: 63.87 ± 4.82 years). Data are summarized in Table 1.

The ALS population, sampled from January 2016 to December 2019, comprised newly diagnosed (ND) ALS patients (7 females and 20 males; mean age ± SD: 64 ± 7.8 years), hospitalized at the Neurology Unit Clinic of the University Hospital of Sassari and long-survivor (LS) ALS patients (10 females and 8 males; mean age ± SD: 65.8 ± 10.4 years) reported by primary doctors and doctors in the Sassari local district.

Peripheral venous blood samples were collected in a K2-EDTA tube. Whole blood was gently layered over an equal volume of Ficoll (Sigma-Aldrich, St. Louis, MO, USA) in a 15 mL tube and centrifuged for 20 min at 1800 RPM without brake. The plasma contained in the uppermost layer was collected by pipetting and tested for the presence of Abs against TDP-43- and HERV-K-env-derived epitopes. All subjects gave their informed consent for inclusion before they participated in the study. The study was conducted in accordance with the Declaration of Helsinki, and the protocol was approved by the Ethics Committee of ALS 1 Sassari (2149/CE).

### 2.2. Peptides

The peptides HERV-K-env-su_(20–38)_ (derived from HERV-K env surface protein, UniprotKB, accession number: O42043), TDP-43_(258–271)_, TDP-43_(398–411)_, and TDP-43_(398–411)_P (derived from TDP-43 protein, UniprotKB, accession number: Q13148) were designed using the Immune Epitope Database and analysis resource (IEDB) and synthesized at > 95% purity (LifeTein, South Plainfield, NJ 07080, USA). The IEDB software predicts regions of proteins that are likely to be recognized as epitopes in the context of a B-cell response. All peptides were dissolved in DMSO and stored at −80 °C in single-use aliquots (10 mM) (Table 2).

### 2.3. Enzyme-Linked Immunosorbent Assay (ELISA)

Indirect ELISA was performed to detect specific Abs against HERV-K-env-su_(20–38)_, TDP-43_(258–271)_, TDP-43_(398–411)_, and TDP-43_(398–411)_P epitopes. Ninety-six-well Nunc immuno-plates were incubated overnight at 4 °C with a solution 0.05 M of carbonate-bicarbonate, pH 9.5 (Sigma-Aldrich, St. Louis, MO, USA) and the respective peptides at 10 µg/mL. The plates were incubated for 1 h at room temperature in a blocking solution with 5% non-fat dried milk (Sigma-Aldrich, St. Louis, MO, USA) and phosphate-buffered saline (PBS) and washed twice in a solution with 0.05% Tween-20 and PBS (PBS-T). The plasma samples were added at 1:100 concentration and incubated for 2 h. After this, each plate was washed five times in PBS-T and incubated for 1 h at room temperature (RT) with 100 µL of PBS and alkaline phosphate-conjugated goat anti-human IgG polyclonal antibody (1:1000, Sigma-Aldrich, St. Louis, MO, USA). After another washing step in PBS-T, plates were incubated in a dark environment for 8 to 10 min in milli-Q water and p-nitrophenyl phosphate (Sigma-Aldrich, St. Louis, MO, USA), and an absorbance of 405 nm was recorded using a SpectraMax Plus 384 microplate reader (Molecular Devices, Sunnyvale, CA, USA). Each sample was run in duplicate, and normalization was performed with the positive control included in each assay. Background activity was calculated as the mean signal of an immobilized peptide with secondary Ab. Results are expressed as the means of duplicates of 405 nm optical density (OD) values.

### 2.4. Statistical Analysis

All data were analyzed using GraphPad Prism 8.2.0 software (GraphPad Software, San Diego, CA, USA). A Mann–Whitney U test was used to analyze non-parametric data and compare differences among two groups. Kruskal–Wallis and Dunn’s post hoc tests were carried out to compare differences among three groups. Statistically significant difference was set with a *p* < 0.05. Receiver-operating characteristic (ROC) was used to choose the cut-off value to assess the sample positivity, which was consequently tested through Fisher’s exact test. A Spearman correlation test was performed among levels of antibodies to HERV-K env- and TDP-43-derived peptides.

## 3. Results

We enrolled 45 ALS patients and 45 matched HCs in our study. Details of the ALS patients’ clinical information are shown in Table 3.

**Figure 1:**
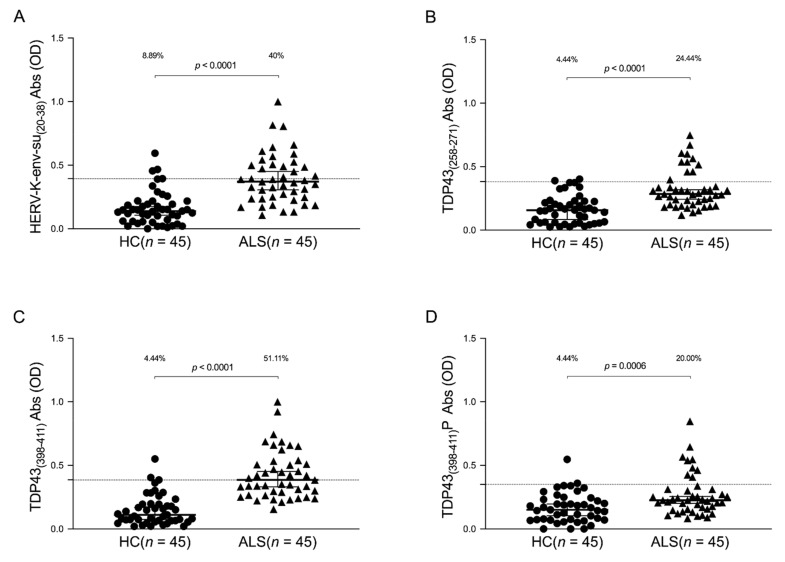
ELISA-based analysis of Abs reactivity against HERV-K- and TDP-43-derived peptides. Plasma samples from ALS patients and HCs subjects were tested against HERV-K-env-su_(20–38)_ (**A**), TDP-43_(258–271)_ (**B**), TDP-43_(398–411)_ (**C**), and TDP-43_(398–411)_P (**D**) peptides. Median and dashed lines represent thresholds used to assess the samples’ positivity. The Mann–Whitney *p-*value and the percentage of positive patients evaluated by Fisher’s exact test are indicated in the upper part of each graph.

The humoral response against HERV-K-env-su_(20–38)_ and three selected epitopes of TDP-43 was evaluated in the plasma of the ALS and HCs groups. A Mann–Whitney U test was performed to compare quantitative values of the antibodies’ OD among groups. Cut-off values were selected by ROC analysis, and the comparison between the percentage of positive and negative samples was performed using Fisher’s exact test. Briefly, Abs against HERV-K-env-su_(20–38)_ were present in 40% of the ALS population, versus 8.89% in the healthy control group (Figure 1A): Mann–Whitney U test, *p* < 0.0001, HCs median = 0.139, 95% CI [0.107, 0.175], ALS median = 0.371, 95% CI [0.305, 0.45]; cut off value of 0.393; Fisher’s exact test, *p* = 0.0011; and AUC = 0.86. We also observed a remarkable Abs response against TDP-43 epitopes in the Sardinian population. In particular, 24.44% of ALS patients showed Abs against TDP-43_(258–271)_, whereas only 4.44% in the healthy group showed a humoral response against the same peptide (Figure 1B): Mann–Whitney U test, *p* < 0.0001, HCs median = 0.157, 95% CI [0.084, 0.191], ALS median = 0.284, 95% CI [0.243, 0.318]; cut off value of 0.381; Fisher’s exact test, *p* = 0.013; and AUC = 0.813. The ALS patients showed an increased Abs presence against the TDP-43_(398–411)_ epitope (Figure 1C), with a positivity measured in 51.11% of the ALS group versus 4.44% of HCs with a Fisher’s exact test *p* < 0.0001 (Mann–Whitney U test, *p* < 0.0001, HCs median = 0.11, 95% CI [0.074, 0.168], ALS median = 0.386, 95% CI [0.332, 0.453]; cut off value of 0.385; and AUC = 0.915). Figure 1D shows the antibody response against TDP-43_(398–411)_P; only 4.44% of healthy subjects were positive, whereas in ALS patients, we observed an increased percentage of up to 20.00% (Mann–Whitney U test, *p* = 0.0006, HCs median = 0.151, 95% CI [0.103, 0.195], ALS median = 0.226, 95% CI [0.202, 0.255]; cut off value of 0.35; Fisher’s exact test, *p* = 0.049; and AUC = 0.706).

**Figure 2:**
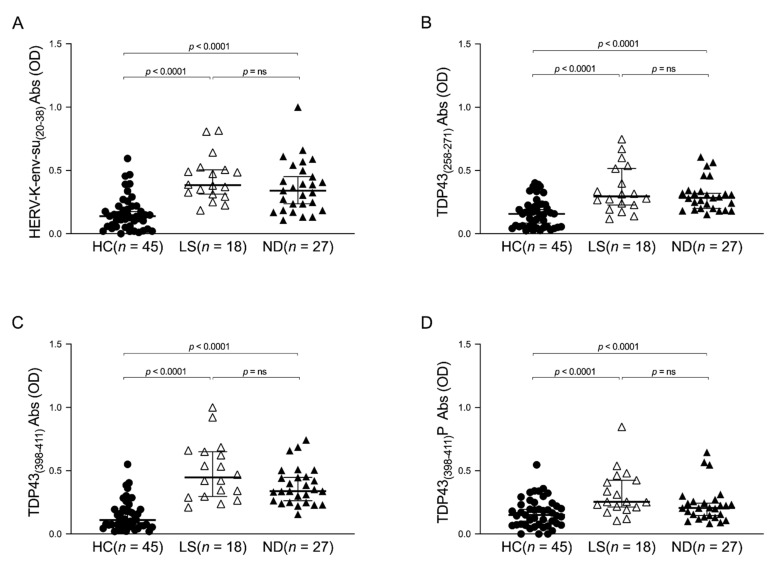
ELISA-based analysis of Abs reactivity against TDP-43- and HERV-K-derived peptides in ALS-ND, ALS-LS, and HCs groups. Plasma samples were tested against HERV-K-env-su_(20–38)_ (**A**), TDP-43_(258–271)_ (**B**), TDP-43_(398–411)_ (**C**), and TDP-43_(398–411)_P (**D**) peptides. A Kruskal–Wallis test and Dunn’s post hoc analysis were performed. Scatter plots represent the median, and the *p*-value is indicated in the upper part of each graph.

In order to investigate a potential difference in the humoral response in the plasma of ALS-ND and ALS-LS patients, the Kruskal–Wallis test and Dunn’s post hoc analysis were performed. The limitation of this analysis is that the three groups (HCs, ALS-LS, and ALS-ND) are not matched by sex due to the small ALS population; it would be very interesting to more deeply understand if statistical differences correlated with sex exist in a larger population. The obtained results highlight a significant difference in HCs compared to ALS-ND and ALS-LS for the peptides derived from TDP-43 and HERV-K envelope protein, while no difference was observed in the human response between ALS-ND and ALS-LS (Figure 2).

**Figure 3:**
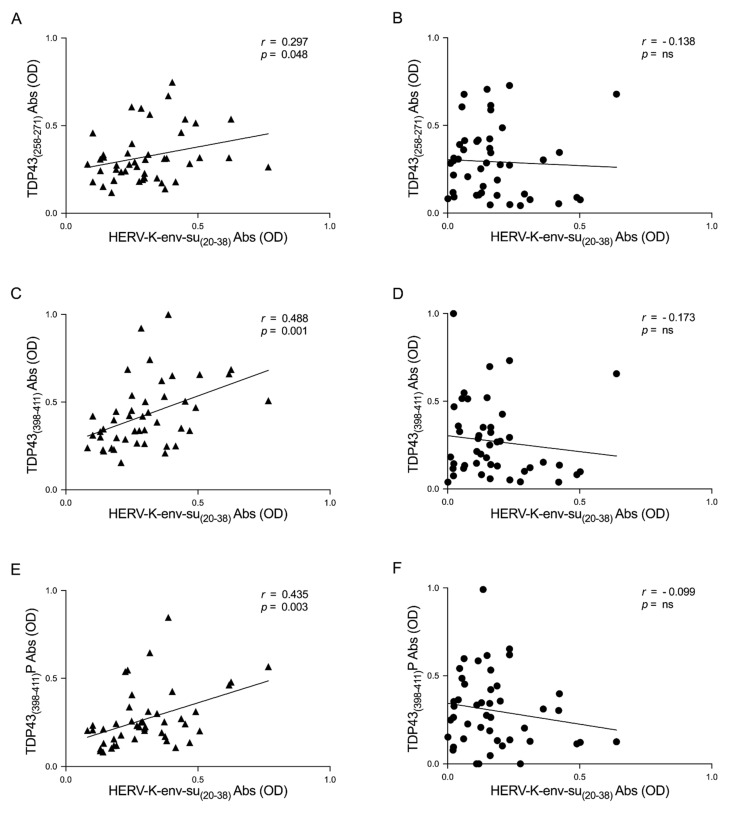
Scatter plots of antibodies to TDP-43, derived peptides, and HERV-K env epitope in ALS populations. The graphs show the correlation between HERV-K-env-su_(20–38)_ and TDP-43_(258–271)_, TDP-43_(398–411)_, and TDP-43_(398–411)_P in ALS patients (**A**,**C**,**E**) and in HCs (**B**,**D**,**F**).

Supplementary analysis was performed to evaluate a possible correlation between TDP-43 and HERV-K humoral response. The results show a correlation between the levels of antibodies and HERV-K and TDP-43 epitopes in ALS patients; these correlations are: HERV-K-env-su_(20–38)_ and TDP-43_(258–271)_ (*r* = 0.297, *p* = 0.048) (Figure 3A), HERV-K-env-su_(20–38)_, and TDP-43_(398–411)_ (*r* = 0.488, *p* = 0.001) (Figure 3C), and finally HERV-K-env-su_(20–38)_ and TDP-43_(398–411)_P (*r* = 0.435, *p* = 0.003) (Figure 3E). The same analysis was performed in HCs to investigate a possible correlation between HERV-K and TDP-43 autoantibody levels. No correlations were observed in HCs; HERV-K-env-su_(20–38)_ and TDP-43_(258–271)_ showed r and *p* values, respectively, of −0.138 and ns (Figure 3B), while HERV-K-env-su_(20–38)_, and TDP-43_(398–411)_ showed an *r* = −0.173 and *p* = ns, (Figure 3D), and finally HERV-K-env-su_(20–38)_ and TDP-43_(398–411)_P showed an *r* = −0.099 and *p* = ns (Figure 3F).

**Figure 4:**
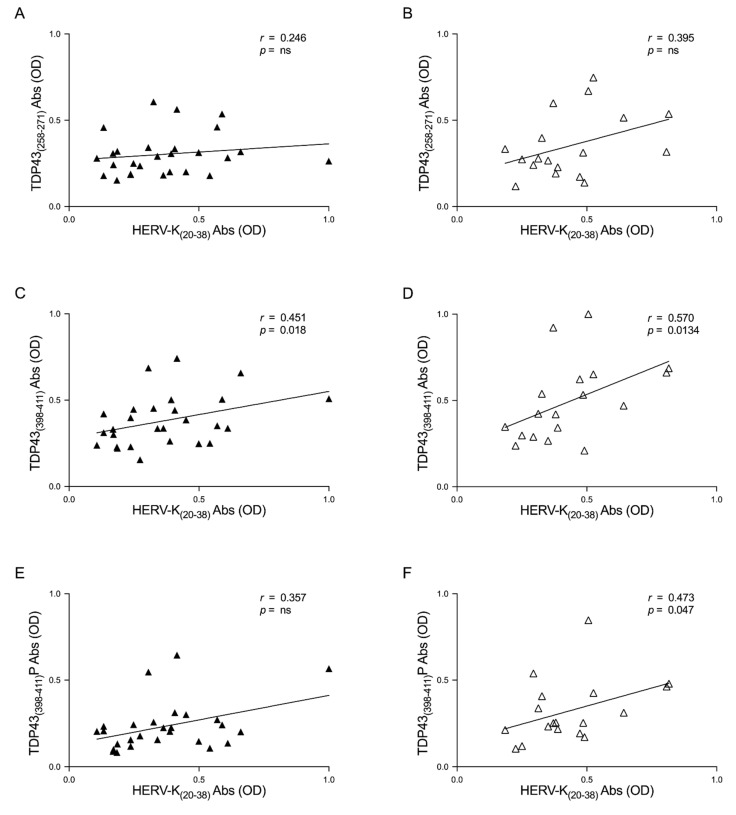
Scatter plots of antibodies to TDP-43-derived peptides and HERV-K env epitope in ALS populations. The graphs show the correlation between HERV-K-env-su_(20–38)_ and TDP-43_(258–271)_, TDP-43_(398–411)_, and TDP-43_(398–411)_P in ALS-ND patients (**A**,**C**,**E**) and in ALS-LS (**B**,**D**,**F**).

In order to deepen this aspect, we investigated the correlation between the humoral response and TDP-43 and HERV-K in patients divided by ALS-ND and ALS-LS. Briefly, we observed positive correlations between the two epitopes in the ALS-ND group, as follows: HERV-K-env-su_(20–38)_ and TDP-43_(258–271)_ (*r* = 0.246, *p* = ns) (Figure 4A), HERV-K-env-su_(20–38)_ and TDP-43_(398–411)_ (*r* = 0.451, *p* = 0.018) (Figure 4C), and lastly HERV-K-env-su_(20–38)_ and TDP-43_(398–411)_P (*r* = 0.357, *p* = ns) (Figure 4E). We observed a positive correlation regarding ALS-LS patients, as follows: HERV-K-env-su_(20–38)_ and TDP-43_(258–271)_ (*r* = 0.395, *p* = ns) (Figure 4B), HERV-K-env-su_(20–38)_ and TDP-43_(398–411)_ (*r* = 0.570, *p* = 0.013) (Figure 4D), and finally HERV-K-env-su_(20–38)_ and TDP-43_(398–411)_P (*r* = 0.473, *p* = 0.047) (Figure 4F).

## 4. Discussion

In this study, we investigated the humoral response directed against specific epitopes of the HERV-K envelope and TDP-43 proteins in human ALS plasma samples. Autoantibodies were found to be significantly higher in ALS patients compared with healthy control samples. These results confirm our previous data and highlight the reactivation of the human endogenous retrovirus in ALS pathology [14].

Likewise, Conti et al., documented increased levels of both anti-TDP-43 Abs and TDP-43 protein in ALS-serum patients compared with healthy controls, motor neuron disease mimics, and Alzheimer’s and frontotemporal lobar degeneration patients, but autoantibodies and protein serum levels failed to correlate [36]. However, recent work has documented a decreased level of anti-TDP-43 Abs in ALS plasma samples [37].

Several studies [38,39] suggest an association between TDP-43 plasma levels and the cerebral accumulation of the protein; however, there is no strong scientific evidence that corroborates this intriguing hypothesis, and further studies are required.

The hypothesis regarding the possible physiological role of autoantibodies in the prevention of excessive accumulation of neuronal inclusions is in agreement with the data reported [40].

Otherwise, the literature has quite clearly proven that the cytoplasmic aggregation of phosphorylated, ubiquitinated, and truncated TDP-43 represents a pathologic hallmark across the clinical spectrum of ALS [6]. The C-terminal region of TDP-43, in particular, is a complex domain, which is essential in the mediation of liquid–liquid phase transitions in the protein, which is central for the biogenesis of various membrane-less organelles such as stress granules, even if makes them prone to misfolding and aggregation [41]. C-terminal fragments (CTFs) are found in cytoplasmic aggregates along with the full-length TDP-43 [6]. Regarding this, our results highlight the increased humoral response directed against selected C-terminal epitopes, including phosphorylated ones, of TDP-43 in ALS patients when compared with HCs (Figure 1). Associating the plasma antibody response with the cerebral accumulation of TDP-43 is particularly intriguing to have an overview of the cerebral accumulation of protein; nevertheless, further investigations are necessary to gain more insight into this aspect.

In order to investigate the role of autoantibodies as a novel biomarker in ALS, we searched for the presence of different autoantibodies in both ALS-ND and ALS-LS patients (Figure 2). The humoral response against the four epitopes selected did not exhibit any statistical difference, indicating that the Abs searched may be present in ALS individuals from the first months after the diagnosis. It would be very interesting to investigate the humoral response in a prospective study looking for autoantibodies at the first symptoms before a definite ALS diagnosis is made.

Different studies have documented the implication of HERV-K in ALS. An altered expression of HERV-K *pol* transcripts in post-mortem brain samples of ALS patients has been confirmed compared to patients with other nervous system diseases and healthy subjects [12]. HERV-K env transgenic cells and mice showed ALS-like pathological and neurological manifestations. Significantly increased levels of anti-HERV-K-env-su Abs were observed by Arru et al., in the serum and cerebrospinal fluid of ALS patients, hinting at the development of a humoral immune response to HERV-K in patients [14].

Furthermore, HERV-K *pol-*gene expression was found to correlate with TDP-43 mRNA in post-mortem brain tissue from patients with ALS [12], and we found a correlation between the humoral response against HERV-K-env-su_(20–38)_ and TDP-43 epitopes.

Interestingly, no correlations have arisen in HCs. This endorses the results obtained in ALS patients and the specific relationship between HERV-K and TDP-43 in pathological conditions (Figure 3). Additional analysis, associated with the duration of the disease, has highlighted a slightly stronger correlation in LS-ALS compared to ND-ALS regarding the Ab levels between TDP-43 and HERV-K (Figure 4). If further studies confirm this observation, that relationship might be considered as a possible marker of ALS progression. With regard to this connection, it would be useful to conduct a prospective study and monitor the patients in ALS evolution.

Currently, the regulatory role of TDP-43 in the HERV-K expression is well known [13], but further and more in-depth studies are needed to understand whether a correlation between the humoral response against TDP-43 and HERV-K exists.

## 5. Conclusions

Our investigation concludes that the epitopes of TDP-43 and HERV-K envelope identified by the Immune Epitope Database (IEDB) are highly immunogenic and recognized by the humoral response found in ALS patients in comparison with the weak response of the healthy control subjects analyzed. We are currently enrolling for a larger study to deepen our hypothesis. In particular, clinical data regarding ALS are going to be collected, in order to point out any putative relationship. It would be interesting to conduct a prospective study to better understand if our results are the consequences of the pathology, or if they are the result of a connection that might exist between TDP-43 and HERV-K env.

Currently, no specific biomarkers are available for the differential diagnosis of ALS, thus diagnostic and prognostic biomarkers for ALS remain a major unmet clinical need, and the results obtained in this work suggest a need for further study in this direction with a larger number of patients and controls.

## Figures and Tables

**Table 1:** Demographic data of investigated groups.

Study Population		Female	Male	Mean Age ± SD
Healthy controls		17	28	63.87 ± 4.82
ALS patients	ND	7	20	64 ± 7.8
	LS	10	8	66.1 ± 10.9

**Table 2:** Epitopes identified in HERV-K-env-su and TDP-43.

	Epitope Position	Epitope Sequence
HERV-K-env-su_(20–38)_	aa 20–38	VWVPGPTDDRCPAKPEEEG
TDP-43_(258–271)_	aa 258–271	SNAEPKHNSNRQLE
TDP-43_(398–411)_	aa 398–411	NGGFGSSMDSKSSG
TDP-43_(398–411)_P	aa 398–411	NGGFGSSMDSK-(PSer)-(PSer)-G

**Table 3:** ALS patients’ clinical data.

ID Patients	Gender	Y/o	Bulbar/Spinal Onset	Disease Duration (Months)	ND-ALS or LS-ALS
ALS 1	M	74	bulbar	1	ND-ALS
ALS 2	M	65	spinal	1	ND-ALS
ALS 3	M	67	spinal	1	ND-ALS
ALS 4	M	66	spinal	1	ND-ALS
ALS 5	M	69	spinal	23	ND-ALS
ALS 6	M	61	spinal	7	ND-ALS
ALS 7	F	58	spinal	1	ND-ALS
ALS 8	M	66	spinal	1	ND-ALS
ALS 9	F	56	spinal	8	ND-ALS
ALS 10	M	78	spinal	1	ND-ALS
ALS 11	F	56	spinal	8	ND-ALS
ALS 12	M	66	spinal	14	ND-ALS
ALS 13	M	44	spinal	16	ND-ALS
ALS 14	M	66	bulbar	9	ND-ALS
ALS 15	F	70	bulbar	3	ND-ALS
ALS 16	F	71	bulbar	6	ND-ALS
ALS 17	M	47	spinal	22	ND-ALS
ALS 18	F	64	spinal	23	ND-ALS
ALS 19	M	61	spinal	4	ND-ALS
ALS 20	M	66	spinal	3	ND-ALS
ALS 21	M	61	spinal	3	ND-ALS
ALS 22	M	62	spinal	20	ND-ALS
ALS 23	M	71	spinal	7	ND-ALS
ALS 24	M	74	spinal	3	ND-ALS
ALS 25	M	71	spinal	3	ND-ALS
ALS 26	M	60	spinal	23	ND-ALS
ALS 27	F	58	spinal	22	ND-ALS
ALS 28	M	71	bulbar	60	LS-ALS
ALS 29	M	68	spinal	36	LS-ALS
ALS 30	M	65	spinal	48	LS-ALS
ALS 31	F	78	spinal	24	LS-ALS
ALS 32	M	59	spinal	240	LS-ALS
ALS 33	F	76	bulbar	132	LS-ALS
ALS 34	F	81	bulbar	48	LS-ALS
ALS 35	F	74	spinal	84	LS-ALS
ALS 36	F	57	bulbar	144	LS-ALS
ALS 37	M	58	spinal	108	LS-ALS
ALS 38	F	48	spinal	48	LS-ALS
ALS 39	F	56	spinal	72	LS-ALS
ALS 40	M	55	spinal	72	LS-ALS
ALS 41	F	65	spinal	48	LS-ALS
ALS 42	F	83	bulbar	96	LS-ALS
ALS 43	M	82	bulbar	96	LS-ALS
ALS 44	F	55	spinal	132	LS-ALS
ALS 45	M	58	spinal	204	LS-ALS
